# Whole genome resequencing data and grain quality traits of the rice cultivar Mahsuri and its blast disease resistant mutant line, Mahsuri Mutant

**DOI:** 10.1016/j.dib.2023.109974

**Published:** 2023-12-18

**Authors:** Siti Amira Adilah Kamarudin, Faiz Ahmad, Nor'Aishah Hasan, Siti Norvahida Hisham, Siti Nurdiyana Yusof, Affrida Abu Hassan, Sobri Hussein, Abdul Rahim Harun, Wan Dalila Wan Chik, Muniroh Md Saad, Mohammad Malek Faizal Azizi, Noraziyah Abd Aziz Shamsudin

**Affiliations:** a Faculty of Applied Sciences, Universiti Teknologi MARA, Cawangan Negeri Sembilan Kampus Kuala Pilah, Kuala Pilah 72000, Negeri Sembilan, Malaysia; b Agrotechnology and Biosciences Division, Malaysian Nuclear Agency, Bangi, Kajang 43000, Selangor, Malaysia; c Department of Biological Sciences and Biotechnology, Faculty of Science and Technology, Universiti Kebangsaan Malaysia, Bangi 43600, Selangor, Malaysia; d Seed Bank Unit, Natural History Museum, Faculty of Science and Technology, Universiti Kebangsaan Malaysia, Bangi 43600, Selangor, Malaysia

**Keywords:** Oryza sativa L., Single nucleotide polymorphisms (SNPs), Rice genomics, Next-generation sequencing (NGS), Bioinformatics

## Abstract

In Malaysia, rice mutant varieties that are genetically altered to confer resistance against blast disease have been substantially developed through mutational breeding program. However, due to the limited accessible information on the mutant lines, mutant gene variants corresponding to the disease resistance and other useful agronomic traits are yet to be exploited. Here, we conducted whole genome re-sequencing of blast resistance with kernel elongation traits in mutant line, Mahsuri Mutant (87,639,446 bp raw reads), and its parental line, Mahsuri (85,156,783 bp raw reads) using Illumina Novaseq 6000 sequencing platform with 30x sequencing coverage. The generated genome sequences are aimed to facilitate the discovery of causal mutation and single nucleotide polymorphisms (SNPs) related to the intended traits. The identified SNPs can be further employed to develop allele-specific SNP molecular markers to locate the mutant gene regions. The NGS data obtained (FASTQ format) of the parental and mutant lines have been deposited in the National Center for Biotechnology Information (NCBI) database under sequence read archive (SRA) xwith accession numbers SRR24388814 (Mahsuri) and SRR22952097 (Mahsuri Mutant) respectively.

Specifications Table

**Table:** 

Subject	Agricultural and biological sciences
Specific subject area	Plant sciences and genomics
Data format	Clean raw reads (FASTQ)
Type of data	Genome sequence data of Mahsuri Mutant and the parent, Mahsuri rice varieties
Data collection	Genomic DNA was extracted from young two-week old seedlings of each rice genotype viz. Mahsuri and Mahsuri Mutant using QIAGEN® DNeasy Plant extraction kit.
Data source location	Institution: Malaysia Nuclear Agency City/Town/Region: Kajang, Selangor Country: Malaysia Latitude and longitude (and GPS coordinate) for collected sample/data: 2.9108° N, 101.7705° E
Data accessibility	The raw reads of the parental and mutant lines are hosted at National Center for Biotechnology Information (NCBI) (https://www.ncbi.nlm.nih.gov/). 1) Repository name: WGS Mahsuri variety Sample: Mahsuri Bioproject:PRJNA965822 NCBI BioSample: SAMN34504494 Data identification number: SRR24388814 [1] Direct URL to data: https://trace.ncbi.nlm.nih.gov/Traces/?view=run_browser&acc=SRR24388814&display=data-access 2) Repository name: Whole genome sequencing Sample: Mahsuri Mutant Bioproject: PRJNA917324 NCBI BioSample: SAMN32536764 Data identification number: SRR22952097 [2] Direct URL to data: https://trace.ncbi.nlm.nih.gov/Traces/?view=run_browser&acc=SRR22952097&display=data-access

## 1 Value of the Data

• The whole genome re-sequencing data of the local mutant rice variety, Mahsuri Mutant and its parent, Mahsuri facilitates the identification of causal mutant genes and their candidate single nucleotide polymorphisms (SNPs) associated with blast disease resistance and kernel elongation traits. Discovery of the genomics and genetics underpinnings of these varieties are beneficial for mutational studies on the Malaysian rice germplasm.
• The causal SNPs can be utilized as breeding resources and further developed as molecular markers to locate the mutant gene regions in rice genome beneficial for genetic improvement programs of the local rice varieties.
• The genome data can be accessed by rice researchers for genetic mapping, SNP calling and evolutionary study to diversify beneficial mutant alleles responsible for controlling blast disease resistance and kernel elongation trait.

## 2 Data Description

In this manuscript, we report the whole genome resequencing (WGRS) data of Mahsuri Mutant rice variety and its parental line, Mahsuri rice variety using high-throughput Illumina Novaseq 6000 sequencing platform at coverage depth of 30x, with generated 150 bp of paired-end reads. The raw data obtained constitutes a sum of an approximate 172.80 Mb raw reads from the entire genome sequences of two aforementioned rice genotypes. After passing through filtering pipelines to eliminate low-quality and contaminated data, a total of approximately 161.67 Mb clean reads were recovered. These clean long-read outputs can be utilised for further data analysis viz. sequence mapping and SNP calling. In this work, the respective genome sequences of Malaysian rice genotypes, Mahsuri Mutant and the parent, Mahsuri were deposited at the National Centre for Biotechnology In-formation (NCBI) (https://www.ncbi.nlm.nih.gov/) under the SRA accession numbers which are SRR22952097 (Mahsuri Mutant) and SRR24388814 (Mahsuri). The summary of the data obtained from re-sequencing approach on these two genotypes are shown in Table 1.

**Table 1:** Data summary of the rice mutant line, Mahsuri Mutant and its parental line, Mahsuri.

Genotype	Traits	Total raw nucleotides (bp)	Total clean nucleotides (bp)	NCBI SRA accession number
Mahsuri Mutant	Resistant to blast disease and possesses elongated kernel	87,639,446	85,156,783	SRR24388814
Mahsuri	Susceptible to blast disease	85,156,783	76,511,683	SRR22952097
Total		172,796,229 ∼172.80 Mb	161,668,466 ∼161.67 Mb	

Utilization of resistance genes in rice has been a premier effort to develop resistant high-yielding cultivars against biotic stresses, particularly rice diseases. Blast disease is introduced as one of the most catastrophic diseases caused by the fungus *Magnaporthe oryzae* to adversely diminish seasonal total yield [6]. Blast resistance trait is governed by single or multiple resistance genes, and the interaction of these genes can potentially increase the genetic variability in the rice genome to become durable against blast. On the other hand, consumers’ preferences for agronomic characteristics such as grain appearance, size, shape, and aroma are also taken into consideration for rice quality improvement. Among these traits, a high kernel elongation ratio is highly preferred by Malaysians, and this trait has high market acceptability and price [5,7]. These advances can be achieved by researchers through mutational breeding, which employs the usage of mutagens to produce agronomically favoured rice that also confers resistance against diseases. *Oryza sativa* L. cv Mahsuri is a famous traditional variety in Malaysia initially introduced as a hybrid product of an interracial cross between the *japonica* and *indica* rice varieties executed by the United Nations Food and Agriculture Organization (FAO) [8]. The program had successfully produced Mahsuri that carried high yielding, was durable to chemical fertilizers and blast disease resistance traits, however, after several years Mahsuri was found to be susceptible and underperforming during blast outbreaks [8,9]. Therefore, induced mutation was performed towards Mahsuri leading to the development of its mutant line, Mahsuri Mutant. Mahsuri Mutant possesses desirable blast resistance trait referring to the preliminary data exhibited in Table 2 and better kernel elongation ratio in cooked rice proposing good eating quality with improved physicochemical properties shown in Table 3 [10]. These data will serve a crucial role in evaluating molecular markers, specifically SNPs and microsatellites from the rice genome, and in advancing rice genotypes genetically. Therefore, further experiments implemented in the future will provide genetic information for evolutionary study.

**Table 2:** Disease scoring of Mahsuri Mutant and its parent, Mahsuri for rice blast artificial screening.

Genotype	0–9 scale	Disease severity	Host response
Mahsuri Mutant	1	Small brown specks of pin-point size or larger brown specks without sporulating center	Resistant (R)
Mahsuri	6	Typical susceptible lesions of 3 mm or longer infecting 11–25 % of the leaf area	Moderately susceptible (S)

The disease severity is scored following the IRRI Standard Evaluation System (SES) [3].

**Table 3:** Physicochemical properties of the rice mutant line, Mahsuri Mutant and its parental line, Mahsuri.

Genotype	Amylose content (%)	Kernel elongation ratio	
		Range	Mean
Mahsuri Mutant	26.1 (high)	2.00–2.31	2.15
Mahsuri	25.4 (high)	1.55–1.86	1.73

The table is modified from Table 3 from the articles referred [4,5].

## 3 Experimental Design, Materials and Methods

### 3.1 Materials

Seeds of the parent line, Mahsuri, were retrieved from Rice Genebank of Malaysian Agricultural Research and Development Institute (MARDI) while the seeds of gamma irradiated mutant line, Mahsuri Mutant were obtained from Malaysia Nuclear Agency (MNA).

### 3.2 DNA extraction, DNA quantification and quality control

Genomic DNA of both rice genotypes, Mahsuri and Mahsuri Mutant, were extracted from young leaves of 14 to 21 days old rice seedlings. DNA extraction was carried out using QIAGEN DNAeasy Plant Extraction Kit by following the manufacturer's protocol (Qiagen, Germany). DNA quantification and qualification was executed immediately using Nano Drop spectrophotometer (Thermo Scientific, USA). DNA integrity was observed by running standard gel electrophoresis in 1 % agarose gel.

### 3.3 DNA library preparation and sequencing

The extracted DNA samples were subjected to downstream processes conducted by the sequencing service provider, Novogene Bioinformatics Technology Co. Ltd. (Beijing, China). Sequencing library was prepared with the addition of read length of 150 bp at both ends using Illumina DNA Prep following the manufacturer's instruction (Illumina, USA). Illumina Novaseq 6000 sequencing platform with 30x coverage depth was utilized to conduct paired-end sequencing on the prepared library.

## Limitations

None.

## Ethics Statement

The authors declare that this work involve neither human subjects nor animal experiments. This manuscript is an original work and has not been published elsewhere.

## CRediT authorship contribution statement

**Siti Amira Adilah Kamarudin:** Writing – original draft. **Faiz Ahmad:** Investigation, Writing – review & editing. **Nor'Aishah Hasan:** Writing – original draft, Validation, Writing – review & editing. **Siti Norvahida Hisham:** Investigation. **Siti Nurdiyana Yusof:** Investigation. **Affrida Abu Hassan:** Validation, Writing – review & editing. **Sobri Hussein:** . **Abdul Rahim Harun:** Validation, Supervision. **Wan Dalila Wan Chik:** Writing – review & editing. **Muniroh Md Saad:** Writing – review & editing. **Mohammad Malek Faizal Azizi:** Writing – review & editing. **Noraziyah Abd Aziz Shamsudin:** Writing – review & editing, Validation, Supervision.

## Data Availability

Whole genome sequencing (Original data) (Sequence Read Archive (SRA)) WGS Mahsuri variety (Original data) (Sequence Read Archive (SRA))
